# Association of Cellulitis With Obesity: Systematic Review and Meta-Analysis

**DOI:** 10.2196/54302

**Published:** 2024-08-20

**Authors:** Kimi Gabriella Taira, Madelyn Wang, William Guo, Olivia Kam, Tara Kaufmann

**Affiliations:** 1 Renaissance School of Medicine Stony Brook University Stony Brook, NY United States; 2 Mayo Clinic School of Graduate Medical Education Rochester, MN United States; 3 Department of Dermatology Stony Brook University Hospital Stony Brook, NY United States

**Keywords:** cellulitis, obesity, overweight, systematic review, meta-analysis, skin infection, body mass index, BMI

## Abstract

**Background:**

Cellulitis is a bacterial skin infection that tends to recur. Previous studies have identified several risk factors that may contribute to its pathogenesis. Obesity is an increasingly prevalent worldwide disease that has been associated with skin and soft tissue infections.

**Objective:**

The aim of our systematic review and meta-analysis was to investigate the association of cellulitis with obesity.

**Methods:**

The Ovid MEDLINE, Embase, Cochrane Central Register of Controlled Trials, and Web of Science databases were searched for the relevant studies from the inception of each respective database to March 13, 2021. Case-control, cross-sectional, or cohort studies that examined the odds or risk of increased BMI in patients with cellulitis were included. This study was carried out in accordance with the PRISMA (Preferred Reporting Items for Systematic Reviews and Meta-Analyses) guidelines. The Newcastle-Ottawa scale (NOS) was used to evaluate the risk of bias in included studies.

**Results:**

In total, 9 case-control studies were included in our quantitative meta-analysis with a total of 68,148 study participants. A significant association was found between cellulitis and obesity (pooled odds ratio [OR] 2.67, 95% CI 1.91-3.71). No significant association was observed between cellulitis and being overweight (pooled OR 1.69, 95% CI 0.99-2.88). Patients with cellulitis were also found to have 1.63-fold increased odds of being male (pooled OR 1.63, 95% CI 1.12-2.38).

**Conclusions:**

Our findings suggest that cellulitis is significantly associated with obesity. Maintaining a healthy BMI may be indicated for patients presenting with cellulitis.

## Introduction

Cellulitis is an acute skin infection commonly caused by the gram-positive bacteria *Staphylococcus aureus* and *Streptococcus* species [1]. Cellulitis results from the entry of bacteria through a breached epidermis into the dermal and subcutaneous layers of the skin, causing an infection that most frequently affects the lower limb, but may involve any part of the body [2]. The clinical findings that characterize cellulitis include erythema, warmth, edema, and pain, in addition to systemic symptoms such as the acute onset (typically within 48 hours) of fever or chills. The severity of cellulitis varies widely, ranging from a well-demarcated erythematous area on the skin with raised borders to a rapidly spreading erythema and possible sepsis [3]. The former definition is widely known as erysipelas and is argued to be a more superficial type of cellulitis. Previous studies have shown that recurrent cellulitis is common, with up to 35%-49% of patients with cellulitis reporting a previous history of cellulitis [4,5]. Several local and general risk factors that may contribute to the development of primary and recurrent cellulitis have been studied, the most prevalent ones being disruption of the skin barrier (such as ulcers, trauma, infection of the toe webs, and dermatomycosis), lymphedema, saphenectomy, history of cellulitis, varicose veins, alcohol abuse, cardiovascular disease, smoking, diabetes, and malignancy [4-12].

Obesity is becoming increasingly prevalent, with over 1.9 billion adults worldwide considered overweight, and of those, 650 million people considered obese [13]. Obesity has been associated with an increased risk of systemic diseases such as cardiovascular disease, type 2 diabetes mellitus, and cancer, as well as infections, particularly of the skin and soft tissue [14-18]. Given the global popularity of various physical exercise programs, it is reasonable to consider the role of physical exercise in decreasing adipose tissue, thereby potentially decreasing the risk of such diseases, including skin and soft-tissue infections [19]. There have been several proposed mechanisms to explain the role by which increased adipose tissue in obese individuals leads to their susceptibility to infections. One is that adipose tissue exerts immunosuppressive effects on the body through key adipokines, and another is that impeded lymphatic flow causes increased bacterial growth, decreased tissue oxygenation, and lymphedema commonly seen in individuals with high BMI [20-22].

There remains some controversy regarding whether systemic factors, such as obesity, play a significant role in the development of cellulitis. Several previous studies have reported no significant association between cellulitis and increased BMI [8,9]; however, other recent studies demonstrate that patients with cellulitis are more likely to be overweight or obese [5,6,10,11]. In 2016, Quirke et al [23] analyzed the risk factors for cellulitis of the lower limbs and found a significant association between being overweight or obese and having cellulitis. However, their review included patients who are obese into their definition of overweight, which may have skewed the association between being overweight and cellulitis toward a more positive direction. As new studies reporting on cellulitis have emerged in the past 5 years [6,10,11], we aim to incorporate the current literature into our analysis to determine whether there is a significant association between cellulitis and obesity. In addition, we aim to explore the potential mechanisms that underlie the association between skin infections and increased BMI and identify possible risk factors that may contribute to the development of cellulitis, such as previous history of skin infections and associated pathogens.

## Methods

### Overview

We conducted a systematic review and meta-analysis of observational studies (including case-control and cohort studies) on the association of cellulitis and obesity. This study was done in accordance with the PRISMA (Preferred Reporting Items for Systematic Reviews and Meta-Analyses) guidelines [24].

The Ovid MEDLINE, Embase, Cochrane Central Register of Controlled Trials, and Web of Science databases were searched for the relevant studies from the inception of each respective database to March 13, 2021. No geographic or language restriction was imposed on our search. Our search strategy is detailed in Table S1 in Multimedia Appendix 1.

### Study Selection

Included studies met the following criteria: (1) observational studies examining the association of cellulitis and BMI, including cross-sectional, case-control, or cohort studies; (2) studies including overweight (25 to 29.9 kg/m² or >120% of the ideal weight as calculated by Lorentz formula) or any class of obesity including class I (30 to 34.9 kg/m²), class II (35 to 39.9 kg/m²), or class III (≥40 kg/m²); (3) the case group was composed of patients with acute or recurrent cellulitis and the control group was composed of individuals without acute or recurrent cellulitis; and (4) the study contained control groups of n>3.

Excluded studies met the following criteria: (1) studies with nonhuman participants, (2) studies not available in English, (3) conference abstracts with no corresponding full-text paper, and (4) studies with case patients who developed cellulitis as a postoperative complication of surgery. Authors MW and KT independently screened the search results to assess their eligibility by reading the titles and abstracts of all citations. Full texts of potentially eligible studies and studies that met the inclusion criteria were read by both authors. Disagreement regarding the eligibility of a study was resolved by a third author, WG.

### Data Extraction and Risk of Bias Assessment

We extracted the following data from the included studies: first author, year of publication, country, study design, diagnosis method of cellulitis, selection criteria of cases and controls, location of cellulitis, definition of obesity/overweight, cultured microbes, and alcohol usage. Quantitative data that were extracted include total number of participants, number of cases and controls, age of participants, percentage of female participants, and univariate and multivariate odds ratios (ORs) with 95% CI on the association of cellulitis with obesity. We used the Newcastle-Ottawa scale (NOS) to assess the risk of bias in included studies [25].

### Statistical Analysis

A pooled OR on the association between cellulitis and obesity, being overweight, as well as sex prevalence, were calculated and depicted in forest plots using the Review Manager software (version 5.4; Cochrane) [26]. Raw patient data were used to calculate available unadjusted pooled ORs. A random effects model of Mantel-Haenszel was used for the OR due to high heterogeneity, as determined by *I*^2^ values greater than 50%. Calculations for pooled multivariate ORs were calculated using an inverse variance method that included adjusted ORs with a random effects model as determined by the *I*^2^ degree of heterogeneity. All calculations were performed with a 95% CI. *P* values less than .05 were considered significant.

## Results

### Search Results

**Figure 1:**
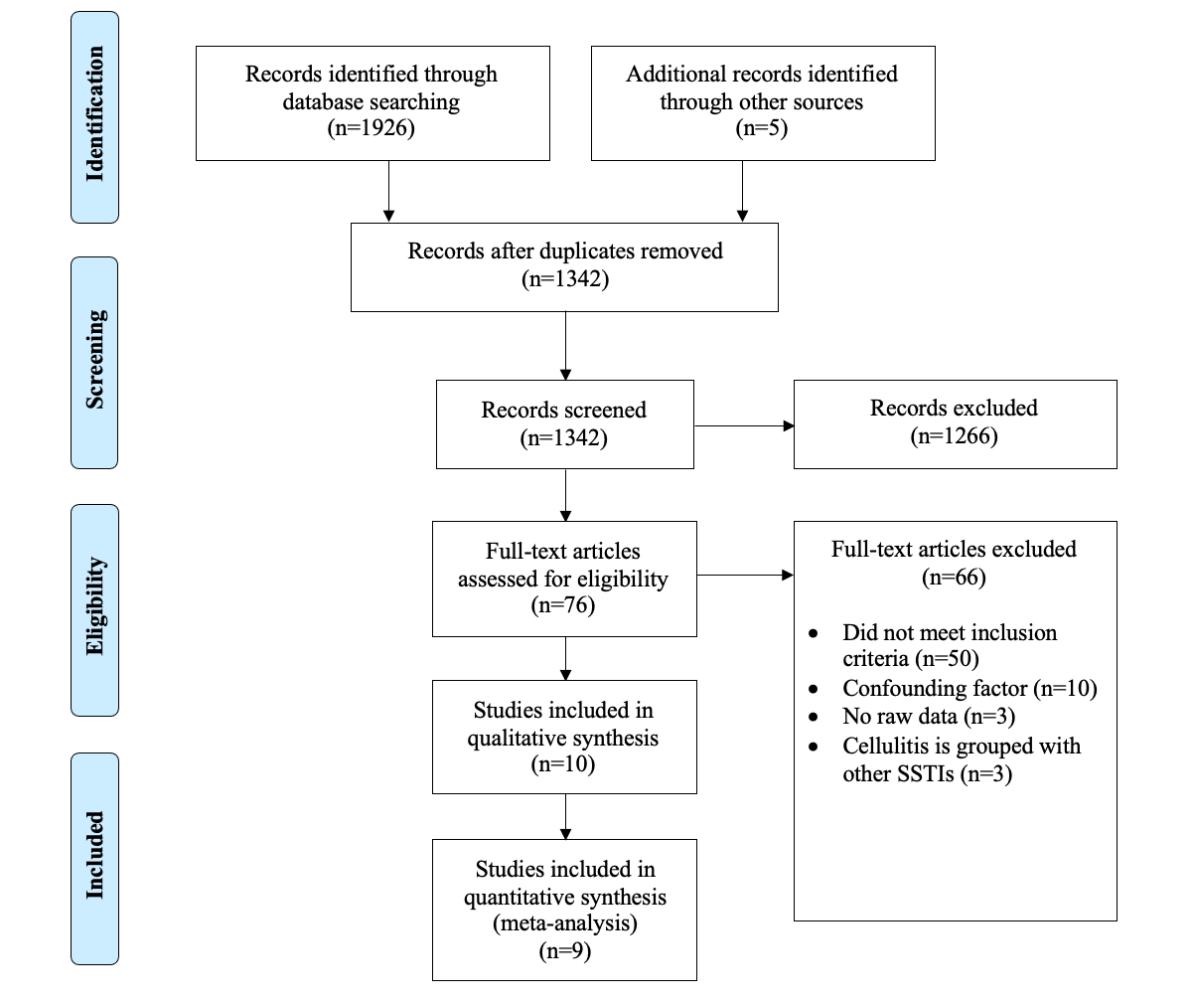
PRISMA (Preferred Reporting Items for Systematic Reviews and Meta-Analyses) study flow chart. SSTI: skin and soft tissue infection.

The PRISMA study flow chart is shown in Figure 1. Our search yielded 1342 citations after removing duplicates. An additional 1266 citations were excluded after reading titles and abstracts. After assessing 76 full-text studies, we excluded 66 studies due to reasons, such as studies not meeting our inclusion criteria (n=50), studies containing confounding factors (n=10), studies not providing raw data (n=3), and studies grouping cellulitis together with other skin and soft tissue infections (n=3). A study by Roujeau et al [12] contained adequate patient data for analysis but was ultimately not included in the quantitative meta-analysis for overweight as it did not meet the inclusion criteria of overweight, which is defined as BMI ≥25 and <30 kg/m^2^. The study did not include data on obesity. A total of 8 studies were ultimately included in our meta-analysis on cellulitis and obesity, and 9 studies were included in our meta-analysis on cellulitis and sex.

### Characteristics of Included Studies

A summary of the characteristics of included case-control studies is listed in Table 1. Our meta-analysis incorporated 9 case-control studies consisting of 68,148 total participants. Out of the total, 37,303 participants were patients with cellulitis, and 30,845 participants were individuals who served as controls. In total, 5 studies used controls matched for age and sex; 2 studies used controls matched for age, sex, and location; and 2 studies used controls matched for age, sex, location, and timing of case. In addition, 6 studies used hospitalized patients as controls, 2 studies used community controls, and 1 study had no description regarding controls. The majority of studies used clinical findings to identify case patients except for 1, which used the *ICD-10* (*International and Statistical Classification of Diseases, Tenth Revision*) codes. A total of 7 studies examined cellulitis solely located on the lower limbs. The study locations included Africa (n=2), Australia (n=1), Europe (n=5), and the Middle East (n=1). Additional characteristics of included case-control studies can be found in Table S2 in Multimedia Appendix 2.

**Table 1:** Characteristics of included case-control studies including study design, description of case and control groups, and odds ratios [4-12].

Source	Study design	Case group patients			Control group patients				OR^a^ (95% CI)
		Total	Overweight, n	Obese, n	Total	Overweight, n	Obese, n		
Dupuy et al 1999 [7]	Case-control	167 patients with cellulitis (80 females and 87 males)	68	—^b^	294 hospital controls matched for age, sex, and hospital (140 females and 154 males)	97	—	1.39 (0.94-2.07)	
Roujeau et al 2004 [12]	Case-control	243 patients with cellulitis (50:50 M/F^c^)	152	—	467 hospital controls matched for age, gender, hospital, and date of admission (50:50 M/F)	174	—	—	
Mokni et al 2006 [9]	Case-control	114 patients with cellulitis (1.6 M/F)	64	—	208 hospital controls matched for age, sex, and hospital (79 females and 129 males)	89	—	1.71 (1.08-2.71)	
Björnsdóttir et al 2005 [4]	Case-control	100 patients with cellulitis (29 females and 71 males)	37	39	200 hospital controls matched for age and sex	86	36	2.91 (1.70-5.00)	
Halpern et al 2008 [8]	Case-control	150 patients with cellulitis (78 females and 72 males)	—	47	300 age and sex-matched controls (156 females and 144 males)	—	70	1.50 (0.97-2.32)	
Karppelin et al 2010 [5]	Case-control	90 patients with cellulitis (32 females and 58 males)	—	37	90 community controls matched for age and sex	—	15	3.49 (1.74-7.00)	
Nassaji et al 2016 [10]	Case-control	102 patients with cellulitis (38 females and 64 males)	37	20	102 community controls matched for age and gender (35 females and 67 males)	17	1	24.63 (3.24-187.45)	
Njim et al 2017 [11]	Case-control	61 patients with cellulitis (40 females and 21 males)	—	27	122 hospital controls matched for age and sex (80 females and 42 males)	—	21	3.82 (1.92-7.62)	
Cannon et al 2018 [6]	Case-control	36,276 patients with cellulitis	—	1192	29,062 hospital controls matched for age, sex, location (postcode), and timing of within 1 month of case	—	401	3.06 (2.73-3.43)	

^a^OR: odds ratio.

^b^Not applicable.

^c^M/F: male to female sex ratio.

### Risk of Bias of Included Studies

**Figure 2:**
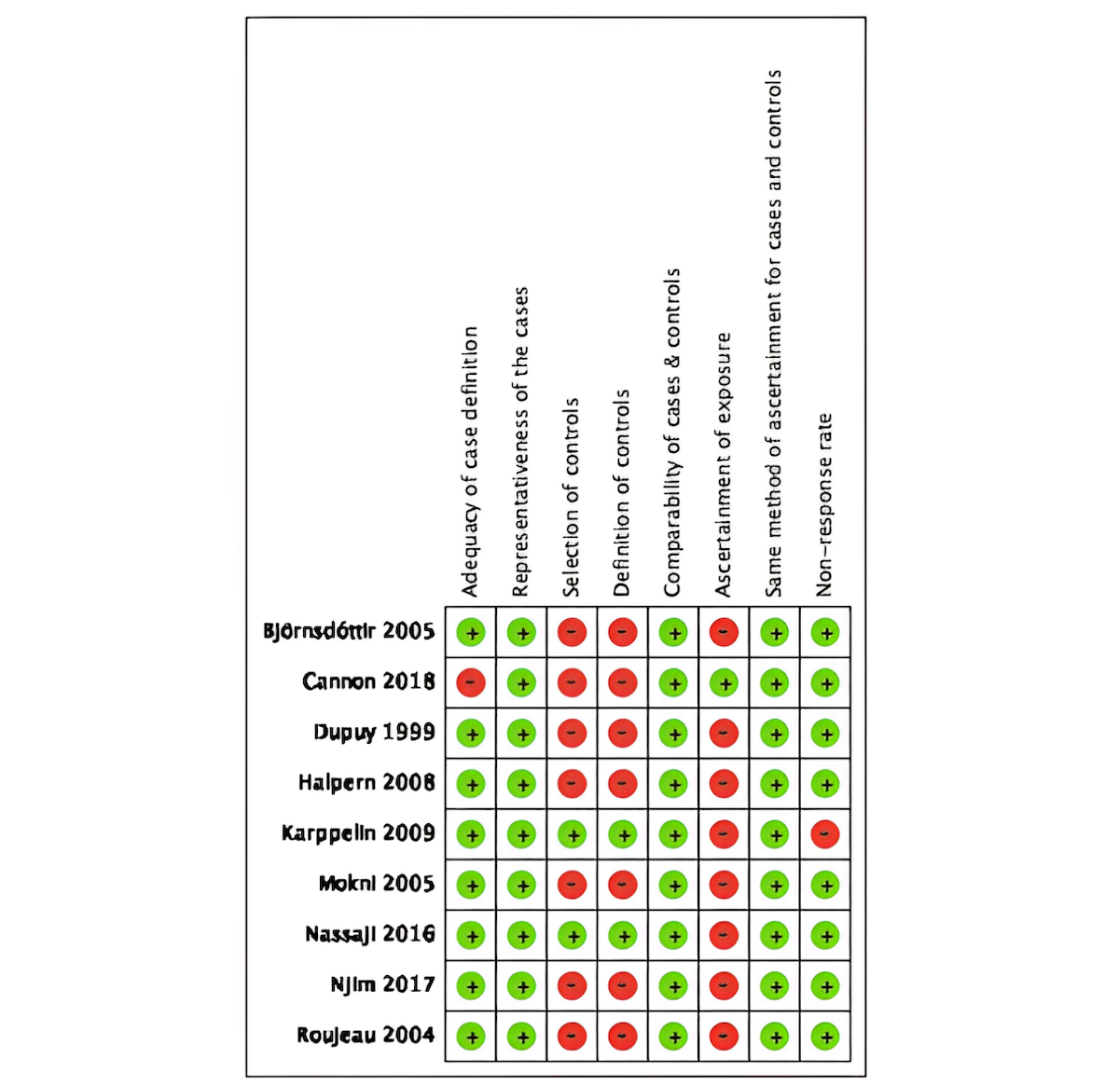
Risk of bias of included case-control studies [4-12]. A green dot denotes low risk of bias and a red dot denotes high risk of bias.

Overall, all 9 studies were rated as low risk of bias according to the NOS. Of the 9 included case-control studies, 8 studies were listed as high risk in ascertainment of exposure. The main reason underlying a high risk in ascertainment of exposure was that most studies did not include a description regarding how obesity status was ascertained among study participants beyond a reporting of ICD codes. In total, 7 case-control studies were listed as high risk of bias in the definition of controls because there was no description regarding the history of disease (cellulitis). In addition, 7 of 9 included case-control studies were listed as high risk in the selection of controls due to the use of hospital controls. We rated the study by Cannon et al [6] as high risk in the adequacy of case definition because ICD diagnosis codes were used to define the case group. All other studies used clinicians to independently validate the definition of case patients. Finally, we rated the study by Karppelin et al [5] as high risk in nonresponse rate because the respondent rate differed between the case group and control group. The risk of bias in included case-control studies is summarized in Figure 2.

### Univariate Pooled Odds Ratio of Cellulitis With Obesity

**Figure 3:**
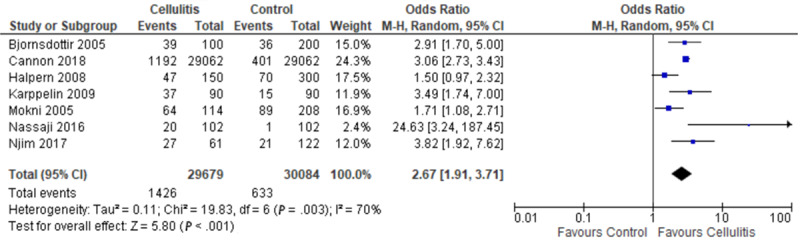
Forest plot on the association of cellulitis with obesity [4-6,8-11]. M-H: Mantel-Haenszel.

Pooling data from 7 case-control studies, our meta-analysis demonstrates a significant association between cellulitis and obesity (pooled OR 2.67, 95% CI 1.91-3.71; Figure 3). With the exception of Halpern et al [8], 6 case-control studies showed a significant association between cellulitis and obesity. There was considerable statistical heterogeneity across all 8 studies (*I*^2^=70%).

**Figure 4:**
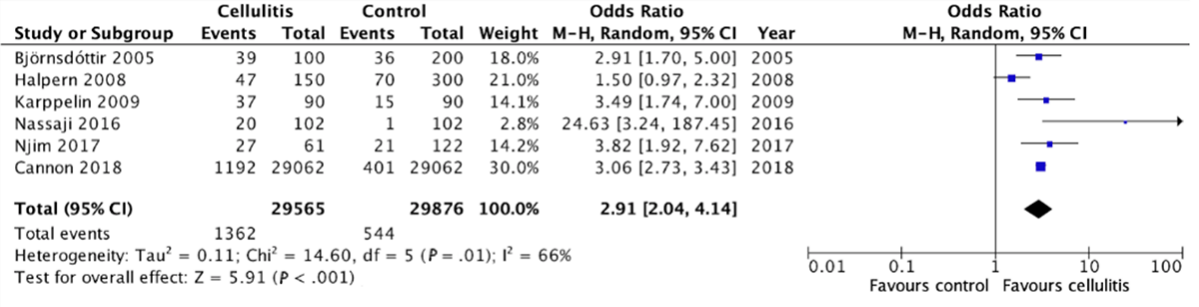
Forest plot on the association of cellulitis with obesity excluding studies using Lorentz formula [4-6,8,10,11]. M-H: Mantel-Haenszel.

A separate meta-analysis was conducted on the 6 case-control studies that used BMI to identify patients who were overweight and obese. The study that was excluded, Mokni et al [9], used Lorentz formula (>120% of ideal body weight) to identify patients with obesity. In this subgroup analysis, we identified increased odds of obesity in association with cellulitis (pooled OR 2.91, 95% CI 2.04-4.14; Figure 4). Substantial statistical heterogeneity was found across the 6 studies (*I*^2^=66%).

### Association of Cellulitis With Being Overweight

**Figure 5:**
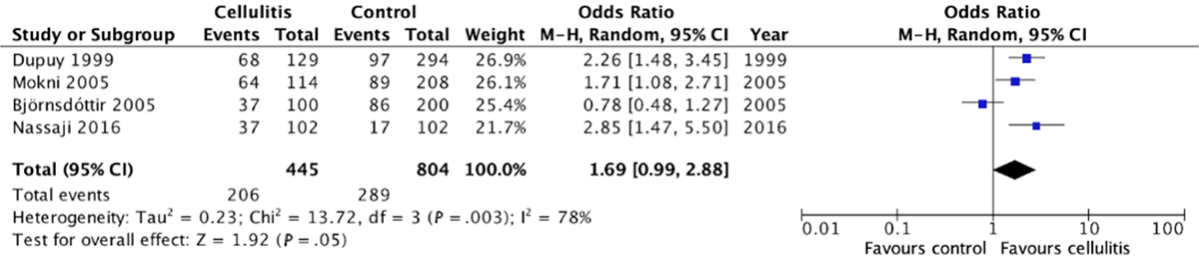
Forest plot on the association of cellulitis with being overweight [4,7,9,10]. M-H: Mantel-Haenszel.

In total, 4 case-control studies provided data on the association between cellulitis and being overweight. Out of these 4, Björnsdóttir et al [4] was the only study that showed no association between cellulitis and being overweight while the other 3 suggested that patients with cellulitis had an increased odds of being overweight. This meta-analysis illustrates that there is no significant association between cellulitis and being overweight (pooled OR 1.69, 95% CI 0.99-2.88; Figure 5). Across the 4 studies, substantial statistical heterogeneity was found (*I*^2^=78%).

### Association of Cellulitis With Sex

**Figure 6:**
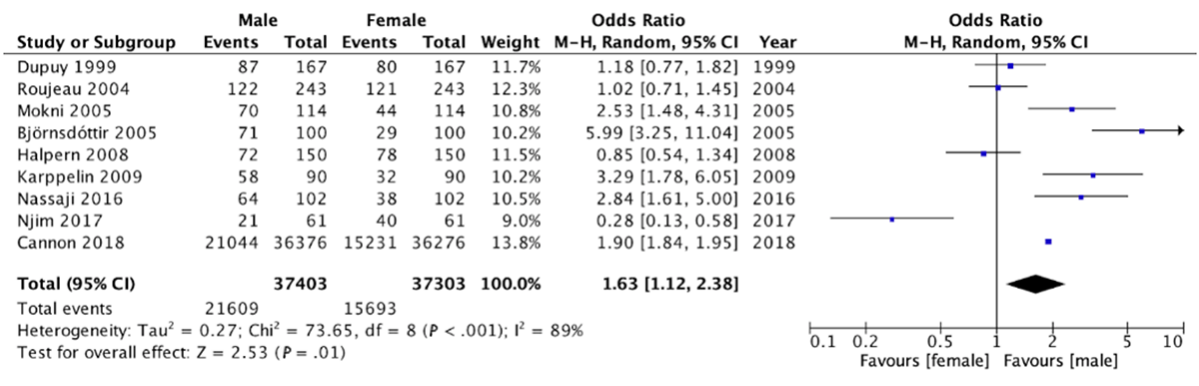
Forest plot on the association of cellulitis with sex [4-12]. M-H: Mantel-Haenszel.

We conducted a meta-analysis on 9 case-control studies that reported data on the association between cellulitis and sex. Of these, 3 case-control studies found no significant association between cellulitis and either sex, while 1 case-control study found a significant association between cellulitis and female sex. The remaining 5 case-control studies demonstrated significantly increased odds of being male and having cellulitis. Substantial statistical heterogeneity was observed across these 9 studies (*I*^2^=89%). When data was pooled from all 9 case-control studies, there was a significant association between cellulitis and male sex (pooled OR 1.63, 95% CI 1.12-2.38; Figure 6).

### Multivariate Analysis on the Association of Cellulitis With Obesity

**Figure 7:**
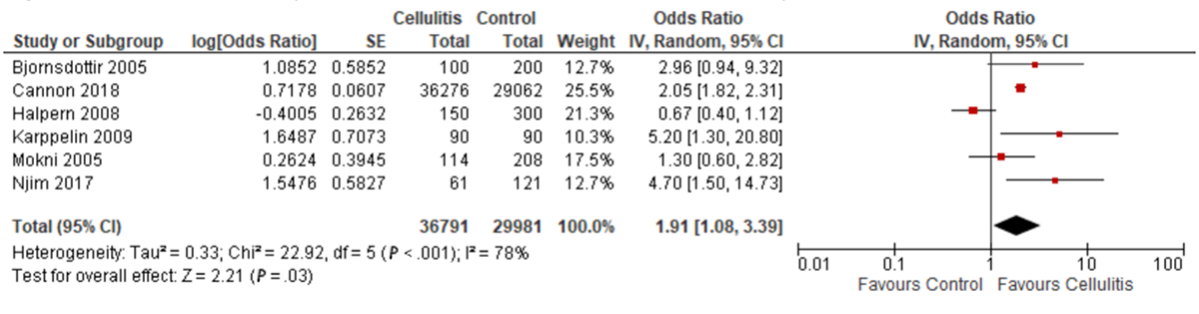
Multivariate analysis on the association of cellulitis with obesity [4-6,8,9,11]. IV: instrumental variable.

**Figure 8:**
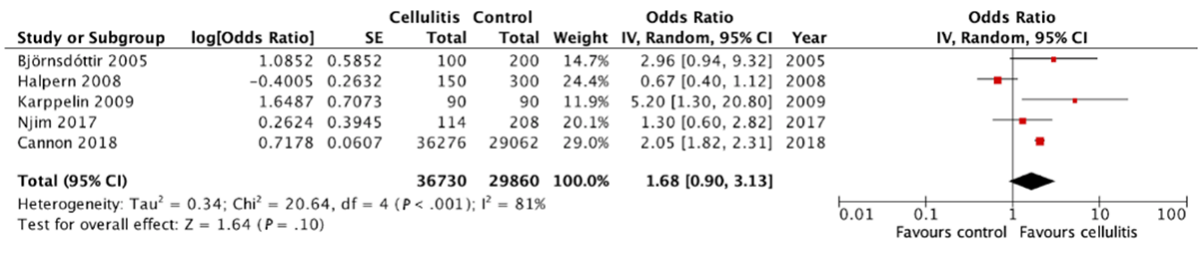
Multivariate analysis on the association of cellulitis with obesity excluding studies using Lorentz formula [4-6,8,11]. IV: instrumental variable.

In total, 6 case-control studies conducted multivariate analyses on the association between cellulitis and obesity. There was significant statistical heterogeneity across these 6 studies (*I*^2^=78%). As shown in Figure 7, the meta-analysis demonstrated a significant association between cellulitis and obesity (pooled OR 1.91, 95% CI 1.08-3.39). When excluding studies that used the Lorentz formula, we did not observe a significant association between cellulitis and obesity on a meta-analysis of the remaining 5 studies (pooled OR 1.68, 95% CI 0.90-3.13; Figure 8). These 5 studies had high statistical heterogeneity (*I*^2^=81%).

## Discussion

### Analysis

In this study, we found that patients with cellulitis are more likely to be obese. The findings from our meta-analysis demonstrate that patients with cellulitis have 2.67-fold increased odds of being obese when compared with controls. Interestingly, our results show that there is no significant association between cellulitis and being overweight. While most studies used BMI to determine overweight and obesity status, Dupuy et al [7] and Mokni et al [9] used the Lorentz formula. When these 2 studies are excluded from the primary analysis, our findings show that patients with cellulitis are still more prone to being obese. Finally, our analysis on the association between cellulitis and sex showed that patients with cellulitis have 1.63-fold increased odds of being male.

In accordance with the NOS, our included studies were ranked as relatively low risk of bias. Of the 9 included case-control studies, 7 studies were scored 6/9, while 2 studies were scored 7/9 and 8/9. The most common reasons for high risk of bias included use of hospitalized patients as controls, absent description regarding how obesity status was obtained, and absent history of the disease (cellulitis).

The results from our analysis on the association of cellulitis with obesity align with those observed by Quirke et al [23] in their previous systematic review. While Quirke et al [23] included studies that exclusively analyzed patients with cellulitis of the leg, 2 of our studies included patients with cellulitis in other areas of the body, such as the upper extremities, trunk, head, face, and genitals [5,10]. Their analysis found a significant association between obesity and having nonpurulent leg cellulitis (OR 2.37, 95% CI 1.39-4.05) [26]. Our results reinforce these findings, as we observed an even stronger association of cellulitis in patients with obesity, with a pooled OR of 2.67 (95% CI 1.91-3.71).

However, our findings differ from Quirke et al [23] regarding the association between cellulitis and being overweight. Their analysis found an association of nonpurulent leg cellulitis in patients who were overweight (OR 1.87, 95% CI 1.26-2.79) while our results demonstrate no significant association between cellulitis and being overweight. This discrepancy may be attributed to our different definition of overweight. Quirke et al defined overweight as a BMI of greater than 25 kg/m², which included patients with obesity in their analysis, and thus may skew the results toward a more positive association. In contrast, we defined overweight as a BMI between 25 and 29.9 kg/m². Due to our narrower definition of overweight, we believe that our findings suggest a more accurate representation of the association between cellulitis and being overweight. However, it is possible that there is a greater association between nonpurulent leg cellulitis and being overweight when compared with cellulitis of other parts of the body. In addition, being overweight may not be associated with cellulitis because overweight individuals have a decreased proportion of adipose tissue compared with obese individuals. Thus, they may experience less immunosuppressive effects from adipokines released by adipose tissue and may have greater lymphatic flow compared with obese individuals [22,27]. Finally, since our study was performed 5 years after the previous analysis, it includes 3 more studies in our analysis on obesity and also includes pooled multivariate and univariate data.

In addition to investigating the association between cellulitis and obesity, we explored certain risk factors that may contribute to the development of cellulitis. One of these factors is having a positive history of cellulitis. Several studies reported this finding. Overall, 183 patients of 685 (26%) with cellulitis had a previous history of cellulitis [4,5,8,10-12]. Karppelin et al [5] also performed an analysis of risk factors between patients with and without a history of cellulitis. They found that patients with a positive history of cellulitis were more likely to have had a previous operation and were likely to stay in the hospital longer than those without a previous history of cellulitis [5]. These patients were also found to have a greater inflammatory response than patients without a history of cellulitis, as demonstrated by a higher peak C-reactive protein level, higher peak leukocyte count, and longer duration of fever after hospital admission.

Some studies also reported on the dermatophytes and bacteria found in interdigital spaces in patients with cellulitis [4,9,11,12]. In cases with foot dermatomycosis, *Trichophyton rubrum* was the most common, followed by *Trichophyton mentagrophytes* and *Epidermophyton floccosum*. In swabs of toe-web spaces with bacteriological culture, beta-hemolytic streptococci and staphylococci were commonly found, while few gram-negative bacilli were identified. Björnsdóttir et al [4] reported that the presence of *Staphylococcus aureus* or beta-hemolytic streptococci in the toe webs was strongly associated with cellulitis. When removing the presence of bacteria from their multivariate model, they found that toe-web dermatophytosis was also strongly associated with cellulitis [4].

Interestingly, some studies described a relationship between cellulitis and patient ethnicity. Halpern et al [8] suggested that patients of White ethnicity are at higher risk of developing cellulitis compared with Asian or Afro-Caribbean patients. In addition, Cannon et al [6] found that Indigenous Australians were more likely than non-Indigenous Australians to develop cellulitis. Explanations for these observed differences are largely speculative, as currently there is little research done in this area. Previous studies have identified that the structure and function of darker skin have properties that may serve as a barrier to bacterial entry, which can influence the risk of developing cellulitis. These properties include greater transepidermal water loss, a greater spontaneous desquamation rate, a lower pH, greater variation in blood vessel reactivity, larger mast cell granules, differences in melanin content and melanosome dispersion, hair structure, fibroblast size, and a more compact stratum corneum, which has greater strength to chemical and mechanical challenges [27-30]. Studies carried out in the United Kingdom have also shown that patients of Caribbean and Asian descent are more likely to seek care from primary care physicians, which may lead to earlier identification and treatment of cellulitis [31-33].

Erysipelas is another acute skin infection, commonly caused by beta-hemolytic group A streptococci, and is often referred to as a superficial type of cellulitis. It affects the upper layer of the skin and is characterized by a warm, slightly painful erythema with a well-demarcated margin, along with an acute systemic response such as fever [34,35]. In contrast, cellulitis is thought to affect the deep dermis and subcutaneous tissue. Erysipelas and cellulitis were historically differentiated because they were thought to be caused by different bacteria; however, a growing body of literature has suggested that they overlap in etiology [34]. Thus, we conducted a review of 5 case series and 1 case-control study which explored the incidence of high BMI in patients with erysipelas. In total, there were 2019 patients with erysipelas, of which 487 (24.1%) were overweight or obese [34-40].

The link between obesity and developing skin infections has been investigated in previous cohort studies [36,41,42]. Among Danish blood donors, Kaspersen et al [36] found that obese men were at a 2-fold increased risk of developing skin and subcutaneous tissue infections. In contrast, Harpsøe et al [41] found that overweight and obese women were at an increased risk of skin and subcutaneous tissue infections. In particular, obese women had a 5-fold increased risk of developing erysipelas. Similarly, a Korean cohort study conducted by Cheong et al [42] demonstrated an increased risk of cellulitis and cellulitis-related hospitalization in metabolically healthy and unhealthy obese men and women. However, it is important to note that Kaspersen et al [36], Harpsøe et al [41], and Cheong et al [42] used select individuals in their cohorts (healthy Danish blood donors, Danish women of reproductive age, and healthy, young, and middle-aged educated Koreans, respectively). Thus, the findings may not be generalizable to other populations. Nevertheless, these results suggest that adiposity itself may be an important contributor to the development of skin infections.

Currently, the pathogenesis underlying the association between obesity and cellulitis is unclear. However, several mechanisms have been proposed to explain the relationship between increased BMI and infection. One possible mechanism involves the effect of excess adipose tissue on the immune system. It is suggested that obesity disrupts the balance between existing adipocytes and immune cells through adipocyte secretion of various adipokines, such as adiponectin and leptin [20-22]. This dysregulation results in impaired macrophage differentiation, chemotaxis, and immune response, all of which may predispose an obese individual to being more susceptible to infections [27]. Obesity may also impair the immune system by contributing to leptin resistance. Leptin plays an important role in regulating immune function through proper signaling in the central nervous system [43,44]. Thus, obese individuals may be more vulnerable to infections because of decreased leptin signaling. Another mechanism provides a more anatomical perspective, citing that the increased skin folds present in obese individuals contribute to decreased blood perfusion of peripheral tissues, thereby increasing the likelihood of abscesses and skin infections [36]. Patient behavior, specifically physical exercise, can play a role in decreasing both body adiposity and hormonal dysregulation, factors that are implicated in the development of cellulitis. Previous studies have shown that exercise that engages both the cardiovascular and the musculoskeletal systems improves anthropometric and body composition, as seen by a decrease in BMI, body fat, and fat-free mass [45]. Recommendation of multicomponent exercise to obese adults can therefore be used as an additional intervention to potentially decrease the likelihood of developing cellulitis.

### Limitations

Our study has several limitations. First, none of our included studies were conducted in Asia or North or South America. Thus, the findings from our meta-analysis may not be generalizable to different ethnic populations, or individuals living in those areas. Second, we were unable to find cohort studies that provided the appropriate data on the association between cellulitis and obesity. More cohort studies are warranted in this area to assess the risk of developing cellulitis in patients who are obese more comprehensively. Finally, the majority of our included studies included hospitalized patients for the case group, control group, or both. The use of hospitalized patients may introduce selection bias, as these patients tend to have more severe sickness, more comorbidities, and older age [5]. Furthermore, community-based studies are needed to investigate cellulitis treated in outpatient settings, as these findings may be more applicable to the general public.

### Future Areas of Research

Future research should be conducted in Asia and North and South America. Given the increased prevalence of obesity in the United States compared with other regions of the world, it would be interesting to explore how studies in the United States may influence the strength of the association between cellulitis and obesity. In addition, it would be beneficial to incorporate cohort studies into future analyses, to strengthen the definitive relationship between cellulitis and obesity. Finally, as cellulitis is also commonly seen in outpatient clinics and in the emergency department, research should focus on the association of cellulitis and obesity in patients from more diverse clinical practices.

### Conclusions

We found that cellulitis is associated with obesity but not with being overweight. Given that the prevalence of obesity continues to increase globally, emphasis on maintaining a healthy BMI may be indicated for patients presenting with cellulitis.

## Appendix

Multimedia Appendix 1 Supplementary tables illustrating search strategy and additional characteristics of included studies.

Multimedia Appendix 2 PRISMA (Preferred Reporting Items for Systematic Reviews and Meta-Analyses) checklist.

## Abbreviations

ICD-10: International and Statistical Classification of Diseases, Tenth Revision
NOS: Newcastle-Ottawa scale
OR: odds ratio
PRISMA: Preferred Reporting Items for Systematic Reviews and Meta-Analyses
