# Supplementary material for: Association of Cellulitis With Obesity: Systematic Review and Meta-Analysis
Source: JMIR Dermatol. 2024 Aug 20;7:e54302. doi: 10.2196/54302 (PMC11372331; doi:10.2196/54302)
Supplement: Multimedia Appendix 1 [file derma_v7i1e54302_app1.docx]

**Table S1.** Search strategy.

| **OVID MEDLINE:** 1946-March 13, 2021. 320 results.   1. exp Cellulitis/ 2. cellulitis.mp. 3. erysipelas.mp. 4. acute cellulitis.mp. 5. acute erysipelas.mp. 6. recurrent cellulitis.mp. 7. recurrent erysipelas.mp. 8. 1 OR 2 OR 3 OR 4 OR 5 OR 6 OR 7 9. humans.sh. 10. exp Obesity/ 11. obesity.mp. 12. obese.mp. 13. obese patient.mp. 14. body mass.mp. 15. body mass index.mp. 16. adiposity.mp. 17. overweight.mp. 18. 10 OR 11 OR 12 OR 13 OR 14 OR 15 OR 16 OR 17 19. 8 AND 9 AND 18 |
| --- |
| **EMBASE:** March 13, 2021. 1,279 results.   1. 'cellulitis'/exp OR 'cellulitis' 2. ‘erysipelas’ 3. ‘acute cellulitis’ 4. ‘acute erysipelas’ 5. ‘recurrent cellulitis’ 6. ‘recurrent erysipelas’ 7. 1 OR 2 OR 3 OR 4 OR 5 OR 6 8. ‘obesity’ 9. ‘obese’ 10. ‘obese patient’ 11. ‘adiposity’ 12. ‘body mass’ 13. ‘body mass index’ 14. ‘overweight’ 15. 8 OR 9 OR 10 OR 11 OR 12 OR 13 OR 14 16. 7 AND 15 |
| **COCHRANE CENTRAL:** 1999-March 8 2021. 1 result.   1. MeSH descriptor: [Cellulitis] explode all trees 2. MeSH descriptor: [Erysipelas] explode all trees 3. #1 OR #2 4. MeSH descriptor: [Obesity] explode all trees 5. MeSH descriptor: [Obesity, Morbid] explode all trees 6. MeSH descriptor: [Adiposity] explode all trees 7. MeSH descriptor: [Body Mass Index] explode all trees 8. MeSH descriptor: [Overweight] explode all trees 9. #4 OR #5 OR #6 OR #7 OR #8 10. #3 AND #9 |
| **Web of Science:** 1900-March 13, 2021. 326 results.   1. TS=(cellulitis) 2. TS=(erysipelas) 3. TS=(acute cellulitis) 4. TS=(acute erysipelas) 5. TS=(recurrent cellulitis) 6. TS=(recurrent erysipelas) 7. #1 OR #2 OR #3 OR #4 OR #5 OR #6 8. TS=(obesity) 9. TS=(obese) 10. TS=(obese patient) 11. TS=(body mass) 12. TS=(body mass index) 13. TS=(adiposity) 14. TS=(overweight) 15. #8 OR #9 OR #10 OR #11 OR #12 OR #13 OR #14 16. 7 AND 15 |
| EndNote: 3/13/21  Imported Records: 1926  Removed Duplicates: 497  After Endnote deduplication: 1429  Number after Rayyan deduplication: 1337  Rayyan Include: 71  Rayyan Exclude: 1266  Rayyan Maybe: 0  Abstract/Title Screen using Rayyan: 5/27/2020  Exclude: 1266  Include: 76  Maybe: 0  Full Text Screen  Exclude: 66  Include: 9 for quantitative analysis (10 for qualitative analysis) |

**Table S2.** Additional characteristics of included case-control studies.

|  |  | **Participant age, years** | |  |  |
| --- | --- | --- | --- | --- | --- |
| **Source** | **Location** | **Case group** | **Control group** | **Obesity/overweight definition** | **Cellulitis definition** |
|  |  |  |  |  |  |
| Dupuy et al 1999 [7] | France | 56.5 (1.8)  mean (SE) | 56.6 (1.1)  mean (SE) | Overweight: > 120% of the ideal weight as calculated by Lorentz’s  formula | Sudden onset (< 24 hours) of a well demarcated cutaneous inflammation, with fever > 38°C or chills |
| Roujeau et al 2004 [12] | Austria, France, Germany, Iceland | 59  mean | 61  mean | Overweight: BMI ≥ 27 | Acute (within 48 hours) onset of signs and symptoms of bacterial cellulitis: consisting of well-demarcated plaque-like cutaneous lesions, characterized by localized erythema, increased warmth and swelling, and associated with fever >38°C and/or chills. |
| Mokni et al 2006 [9] | Tunisia | 45.4 ± 15.7  mean ± SE | 45.0 ± 15.9  mean ± SE | Overweight: > 120% of the ideal weight as calculated by Lorentz’s  formula | Sudden onset (< 24h) of a well demarcated acute dermohypodermatitis considered to be non-necrotizing cellulitis with fever >38° C or chills |
| Björnsdóttir et al 2005 [4] | Iceland | 66.5 (48.8-77.0)  median (IQR) | 66.5 (50-75)  median (IQR) | Overweight: BMI ≥ 25 and <30  Obese: BMI ≥ 30 | Demarcated cutaneous inflammation of sudden onset (over < 72 h) that was associated with fever, chills or leukocytosis (leukocyte count, >10.5 x 10⁹ cells/L) |
| Halpern et al 2008 [8] | United Kingdom | 68.2 ± 1.5  mean ± SEM | 68.2 ± 0.9  mean ± SEM | Obese: BMI >30 | An acute spreading pyogenic inflammation of the dermis and subcutaneous tissues, characterized by a tender, warm, erythematous, swollen leg without sharp demarcation from uninvolved skin |
| Karppelin et al 2010 [5] | Finland | 58 (21-90)  median (range) | | Obese: BMI ≥ 30 | Recent history of acute onset of fever or chills and localized erythema of the skin on one extremity or the typical appearance of a well-demarcated skin lesion on the face, with or without fever or chills |
| Nassaji et al 2016 [10] | Iran | 45.6 ±16.8  mean ± SD | 46.5 ±16.8  mean ± SD | Overweight: BMI 25-29.9  Obese: BMI ≥ 30 | Pain, warmth, edema, erythema without sharp demarcation from uninvolved skin with or without fever |
| Njim et al 2017 [11] | Cameroon | 52 (32.5-74.5)  median (IQR^a^) | | Obese: BMI ≥ 30 | Localized area of lower limb erythema, warmth, oedema, and pain associated with fever > 38 °C and/or chills of sudden onset |
| Cannon et al 2018 [6] | Australia | 52 (34-72)  median (IQR) | NA^b^ | Obese: ICD 10 code E66 (or ICD 9 code 278) | ICD 10th Revision codes (L03.11, cellulitis of the lower limb; L03.9, cellulitis unspecified; L04.3, lymphadenitis of lower limb and A46, erysipelas) |

^a^ICD: international classification of diseases

^b^NA: not available
